# Pump-Free Microfluidic Hemofiltration Device

**DOI:** 10.3390/mi12080992

**Published:** 2021-08-20

**Authors:** Takahiro Ito, Takashi Ota, Rei Kono, Yoshitaka Miyaoka, Hidetoshi Ishibashi, Masaki Komori, Akio Yasukawa, Yoshihiko Kanno, Norihisa Miki

**Affiliations:** 1 Department of Mechanical Engineering, Keio University, 3-14-1 Hiyoshi, Kohoku-ku, Yokohama 223-8522, Kanagawa, Japan; ito.takahiro@keio.jp (T.I.); takashi94@z3.keio.jp (T.O.); rei.kono@keio.jp (R.K.); 2 Department of Nephrology, Tokyo Medical University, 6-7-1 Nishi-Shinjuku, Shinjuku-ku, Tokyo 160-0023, Japan; mitaku380947@gmail.com (Y.M.); kannoyh@tokyo-med.ac.jp (Y.K.); 3 Pre-Clinical Research Center, Tokyo Medical University, 6-1-1 Shinjuku, Shinjuku-ku, Tokyo 160-8492, Japan; ishiba@tokyo-med.ac.jp; 4 Japanese Small Animal Hemodialysis Association, 63-2-7 Nihonbashi-Hongokucho, Chuo-ku, Tokyo 103-0021, Japan; komorin007@yahoo.co.jp; 5 Kamishakujii Animal Hospital, 1-4-13 Sekimachi-Higashi, Nerima-ku, Tokyo 177-0052, Japan; the_laser_man@vets-surg.com

**Keywords:** microfluidic, artificial kidney, hemofiltration, backfiltration, oncotic pressure, transmembrane pressure, artificial organ, animal test

## Abstract

Hemofiltration removes water and small molecules from the blood via nanoporous filtering membranes. This paper discusses a pump-free hemofiltration device driven by the pressure difference between the artery and the vein. In the design of the filtering device, oncotic pressure needs to be taken into consideration. Transmembrane pressure (TMP) determines the amount and direction of hemofiltration, which is calculated by subtracting the oncotic pressure from the blood pressure. Blood pressure decreases as the channels progress from the inlet to the outlet, while oncotic pressure increases slightly since no protein is removed from the blood to the filtrate in hemofiltration. When TMP is negative, the filtrate returns to the blood, i.e., backfiltration takes place. A small region of the device with negative TMP would thus result in a small amount of or even zero filtrates. First, we investigated this phenomenon using in vitro experiments. We then designed a hemofiltration system taking backfiltration into consideration. We divided the device into two parts. In the first part, the device has channels for the blood and filtrate with a nanoporous membrane. In the second part, the device does not have channels for filtration. This design ensures TMP is always positive in the first part and prevents backfiltration. The concept was verified using in vitro experiments and ex vivo experiments in beagle dogs. Given the simplicity of the device without pumps or electrical components, the proposed pump-free hemofiltration device may prove useful for either implantable or wearable hemofiltration.

## 1. Introduction

Hemofiltration is a blood purification therapy, removing water and low molecular weight ions from blood using nanoporous filtration membranes [1]. To enhance the efficiency of hemofiltration, an external pump is often used to augment the transmembrane pressure (TMP) [2]. The amount of filtrate is the product of the filtration coefficient, membrane area, TMP, and time [3,4].

**Figure 1:**
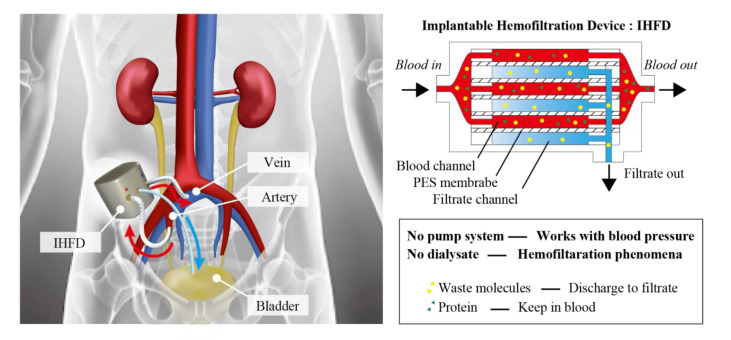
Concept of implantable hemofiltration device (IHFD). The device is connected to artery and vein and removes water and wastes from blood by hemofiltration. The filtrate is brought to bladder and discarded as urine. IHFD works with blood pressure and does not require a pump system. IHFD, which continuously removes water from chronic kidney disease patients, reduces the frequency of their hospital visits, alleviates the water intake restriction, and thus, drastically improves their quality of life.

Our group has been developing implantable blood purification systems to free hemodialysis patients from both frequent hospital visits and strict restrictions on water intake [5]. An implantable system mandates the following considerations in design. First, hemodialysis using the diffusion-based exchange of substances between the blood and the dialysis fluid is unsuitable due to difficulties in storage and pumping of the dialysis fluid. Second, the blood should preferably be driven by blood pressure rather than by pumps that require a power supply and electrical and mechanical components. Based on these considerations, we designed an implantable hemofiltration device (IHFD) (Figure 1). This device consists of multi-layered channels for blood and filtrate that are separated by nanoporous polymer membranes of polyethersulfone (PES). The IHFD is connected to the artery and vein and the pressure difference between the vessels drives the blood flow. No pumps or electrical components are necessary. The blood pressure and the amount of the filtrate varies with time, whereas this variation is acceptable as long as the total amount of the filtrate per day reaches the requirement. The total amount can be controlled by the number of the layers. The filtrate is brought to the bladder and discarded as urine. We consider that this as one of the simplest and most compact designs. A similar design was proposed using nanoporous silicon membranes [6,7]. Of note, a major priority of the IHFD is removing water from blood. If a water volume of 1.5–2.0 L can be removed from the body, the patient can be freed from severe restrictions on water intake. Indeed, removing all the waste material from blood by hemofiltration alone is not feasible; such a task would require the filtration of 150 L of blood each day. Our idea was to combine the IHFD with conventional hemodialysis treatment at a reduced frequency. This hybrid approach should drastically improve quality of life for hemodialysis patients.

Achieving the ultimate goal of developing the IHFD requires us to overcome plenty of challenges. Filtration capacity and blood compatibility need to be investigated over a period of many years. Clinical procedures using an IHFD that include implant surgery, medications, and monitoring need to be designed. Such challenges will be discussed elsewhere in the near future.

**Figure 2:**
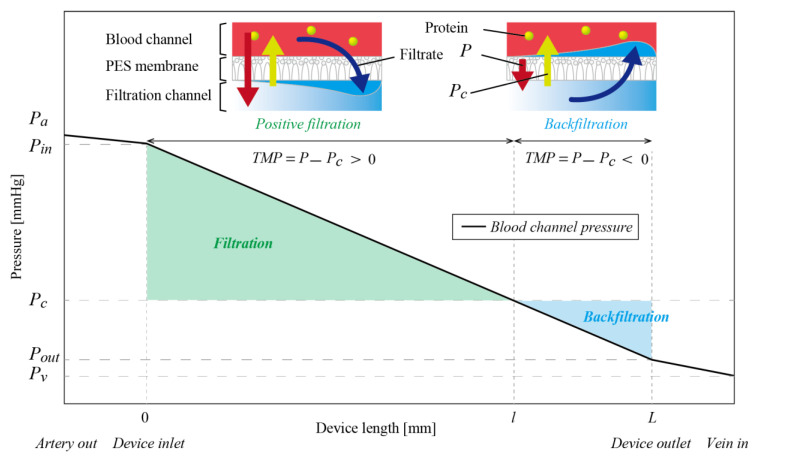
Pressure inside the blood channel with respect to position. The amount of filtrate is determined by the transmembrane pressure *TMP*, representing the difference between blood pressure P and oncotic pressure $P_{c}$. When *TMP* is negative, backfiltration takes place and the total amount of filtrate decreases or even becomes zero. In humans, pressures in the artery and vein, $P_{a}$ and $P_{v}$, are generally 90–130 mmHg and 5–10 mmHg, respectively, and the oncotic pressure $P_{c}$ is 24–33 mmHg for a total protein concentration of 6.7–8.3 g/dL. Therefore, when only the hemofiltration device is connected to the artery and vein, *TMP* is negative and backfiltration takes place in some regions.

In this paper, we discuss a channel design that takes backfiltration into consideration. The pump-free IHFD works using the difference between arterial and venous pressures. Venous pressure depends on position and posture, and is typically around 20 mmHg [8] in normal cases, while arterial pressure is around 80 mmHg. Oncotic pressure, produced by the presence of protein in blood, is around 30 mmHg. Therefore, as shown in Figure 2, TMP decreases along the filtration channels and becomes negative from a certain point. Where TMP is negative, filtrate returns to the blood in a process termed “backfiltration” [9,10]. Backfiltration has been studied in hemodialysis applications in the context of contaminants in the dialysate being brought back to the blood, which is not the case for the IHFD.

**Figure 3:**
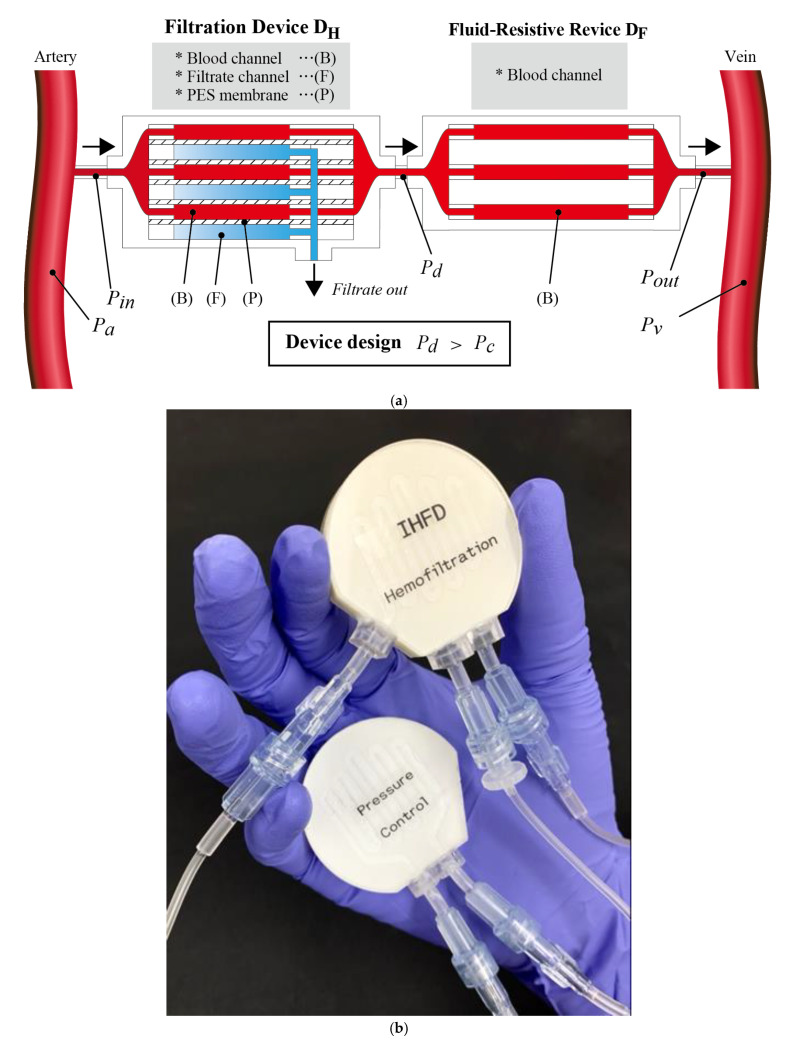
(**a**) Serial connection of hemofiltration device *D_H_* and fluid-resistive device *D_F_*. In this figure, the number of layers for saline (or blood) channels $N_{D_{H}\;}$ and $N_{D_{F}}$ are both 3. When *D_H_* and *D_F_* are designed such that the pressure between devices $P_{d}$ exceeds the oncotic pressure $P_{c}$, no backfiltration takes place. (**b**) Photograph of the serially connected hemofiltration device *D_H_* (“hemofiltration”) and fluid-resistive device *D_F_* (“pressure control”).

Based on this discussion, we designed a microfluidic device between the hemofiltration device and vein to generate a pressure drop and ensure that TMP inside the hemofiltration device always remains positive, as illustrated in Figure 3a. We call this device the fluid-resistive device *D_F_*. In vitro and ex vivo experiments were conducted to verify the concept and determine the design of the hemofiltration devices. Given the simplicity of a device lacking pumps or electrical components, the proposed pump-free hemofiltration device would be usable for either implantable or wearable hemofiltration.

## 2. Device Design and Experimental Method

### 2.1. Device Design

Figure 3a,b shows the design and a photograph of the pump-free filtration device with the hemofiltration device *D_H_* and fluid-resistive device *D_F_*. *D_H_* and *D_F_* comprise multilayered microchannels formed by polyurethane sheets [11,12,13]. They both have identical channel designs, with channel widths of 2 mm and channel heights of 0.4 mm. The channels have 9 turns and the length of one layer is 200 mm (membrane area, 400 mm^2^). *D_H_* has channels for filtration that are separated by the filtering membrane. *D_F_* does not have filtration channels and polyurethane sheets are sandwiched by the filtering membranes. In the experiments, *D_H_* comprised 5 layers for saline solution channels and 5 layers for filtrate. We tested *D_F_* with 1 layer, 5 layers, and 10 layers for the saline solution.

### 2.2. Amount of Filtrate

The filtration coefficient $L_{p}$ is determined as:

(1) $$L_{p}=\frac{q_{f}}{TMP\times A}$$

where $q_{f}$ is the volumetric amount of filtrate per time, TMP is the transmembrane pressure, and A is the effective area for filtration. TMP can be described as:

(2) $$TMP=P-P_{f}-P_{c}$$

where P and $P_{f}$ are the pressures of the blood and filtrate, respectively. $P_{c}$ is the colloidal osmotic pressure, resulting from albumin and other large molecules in the blood [14]. Given the low flow rate of the filtrate and that the filtrate channel is open to ambient conditions, $P_{f}$ was considered negligible in our experiments. $P_{c}$ can reportedly be expressed using the total protein concentration $C_{TP}$ as [15,16,17,18]:

(3) $$P_{c}=2.1C_{TP}+0.16C_{TP}{}^{2}+0.009C_{TP}{}^{3}$$

When TMP is negative, backfiltration takes place. Let us consider a case in which only *D_H_* is tested. Presuming the total length of the *D_H_* is L and TMP becomes zero at length l, the total amount of filtrate per time $q_{total}$ is expressed as:

(4) $$q_{total}=\int_{0}^{l}L_{pf}TMP\left(x\right)wNdx-\int_{l}^{L}L_{pbf}TMP\left(x\right)wNdx$$

where x is the position, $L_{pf}$ and $L_{pbf}$ are the filtration coefficients of filtration and backfiltration, respectively, w is the channel width, and N is the number of the layers. When pressures at the inlet and outlet of *D_H_* are $P_{in}$ and $P_{out}$, respectively, and $P_{in}>P_{c}>P_{out}$, l is expressed as:

(5) $$l=\frac{P_{in}-P_{c}}{P_{in}-P_{out}}L\;$$

When $L_{pf}$, $L_{pbf}$, and $C_{TP}$ are known, $q_{total}$ can be calculated as a function of $P_{in}$ and $P_{out}$.

### 2.3. Hemofiltration Device D_H_ and Fluid-Resistive Device D_F_

When we only connect the hemofiltration device to the artery and vein of the recipient animal, $P_{in}$ and $P_{out}$ are approximately $P_{a}$ and $P_{v}$, as the arterial and venous pressures, respectively. $P_{a}$, $P_{v}$, and $P_{c}$ depend on the animal. However, in our experiments, many cases showed $P_{c}>P_{v}$ when backfiltration took place and the amount of filtrate decreased or no filtrate was obtained (Figure 2). We therefore connected the fluid-resistive device *D_F_* serially with *D_H_* to prevent backfiltration. $P_{d}$ is the pressure inside the channel between *D_H_* and *D_F_*. Pressure losses across the devices, $P_{a}-P_{d}$ and $P_{d}-P_{v}$, are roughly proportional to the flow velocity $v_{D_{H}}$ and $v_{D_{F}}$, as the flow velocity inside *D_H_* and *D_F_*, because the Darcy friction factor is inversely proportional to the Reynolds number under our experimental conditions. Since *D_H_* and *D_F_* are connected serially, volumetric flow rates in *D_H_* and *D_F_* are conserved and the ratio of flow velocities $v_{D_{H}}$ and $v_{D_{F}}$ is $N_{D_{F}}/N_{D_{H}}$, where $N_{D_{H}}$ and $N_{D_{F}}$ are the number of layers of *D_H_* and *D_F_*, respectively. When $P_{a}$, $P_{v}$, $N_{D_{H}}$, and $N_{D_{F}}$ are given, $P_{d}$ is expressed as:

(6) $$P_{d}=\frac{N_{D_{H}}P_{a}+N_{D_{F}}P_{v}}{N_{D_{H}}+N_{D_{F}}}$$

When $P_{d}>P_{c}$, no backfiltration takes place inside *D_H_*. In the experiments, $N_{D_{H}}$ was 5 and $N_{D_{F}}$ values of 1, 5, and 10 were tested.

Without backfiltration, i.e., $P_{d}>P_{c}$, the amount of filtrate can be expressed as:

(7) $$q_{total}=L_{pf}\left(\frac{P_{in}+P_{d}}{2}-P_{c}\right)wLN$$

which was transformed from Equation (4).

### 2.4. Experimental Method

#### 2.4.1. Device Fabrication

The device consists of microchannels, nanoporous filtering membrane, and sealing plates. The filtering membrane is made of PES, using a liquid inversion method to create a thickness of 100 μm [19,20]. PES with a molecular weight of 4800 (Sumitomo Chemical Co., Tokyo, Japan), polyethylene glycol with a molecular weight of 400 (FUJIFILM Wako Chemical Corporation, Osaka, Japan) and dimethylacetamide (DMAc) (FUJIFILM Wako Chemical Corporation) were mixed in ratios of 17.5 wt%, 14.6 wt%, and 67.9 wt%. The solution was then spin-coated onto a glass substrate to create a thickness of 100 μm, then immersed into deionized (DI) water to form a membrane. Note that PES membranes are asymmetric and have nanoporous layers and microporous layers.

**Figure 4:**
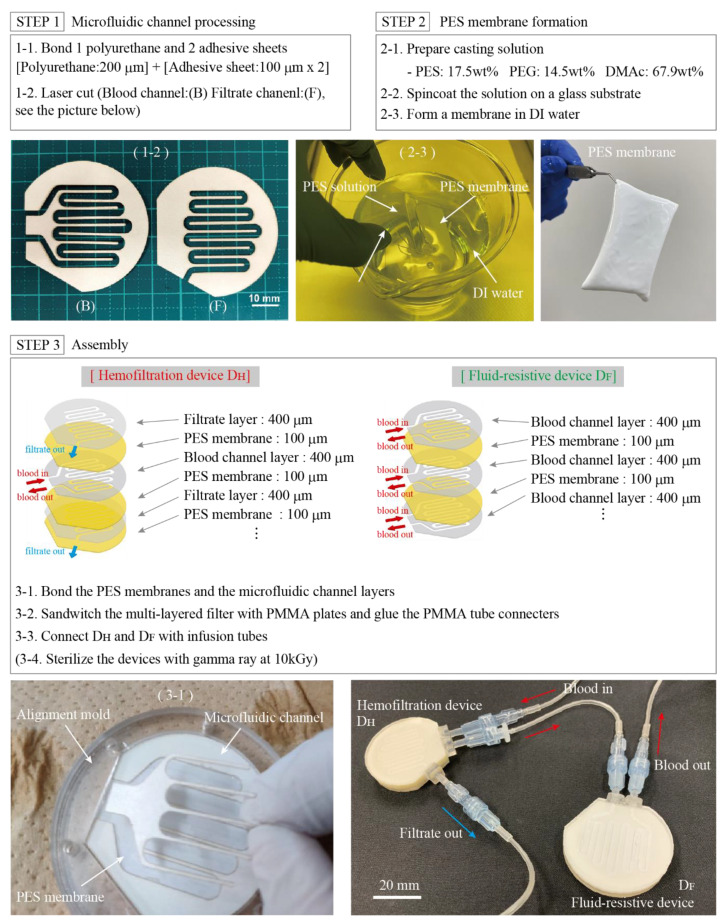
Fabrication process of the hemofiltration device and the fluid-resistive device. Microfluidic channels with adhesives are laser cut to have two patterns, as shown in the Step 1. PES membranes are formed by the liquid inversion method (Step 2). The devices are formed by bonding the channels and PES membranes alternatively, where they are aligned with a jig. PES membranes are known to be asymmetric and have nanopores on one side and micropores on the other side. Note that the blood (or sample) channels contact with nanoporous side of the membrane.

The channels were formed from polyurethane (PU) sheets, 200 μm in thickness (Higress; Sheedom Co., Osaka, Japan) by laser cutting. The sealing plates are made of polymethyl methacrylate (PMMA), 2 mm in thickness. Double-sided adhesive sheets for medical use (1504XL; 3M Japan, Tokyo, Japan) were used for assembly. As shown in Figure 4, the adhesive sheets were applied to both sides of the PU sheets. PES membranes were attached, where the channels for blood contact the nanoporous layer. Connectors to connect the devices to the extension tubes were formed by cutting a 5 mm-thick PMMA sheet, then bonded with medical-use adhesive (Aron Alpha A; Daiichi Sankyo, Tokyo, Japan).

#### 2.4.2. Perfusion System for In Vitro Experiments

**Figure 5:**
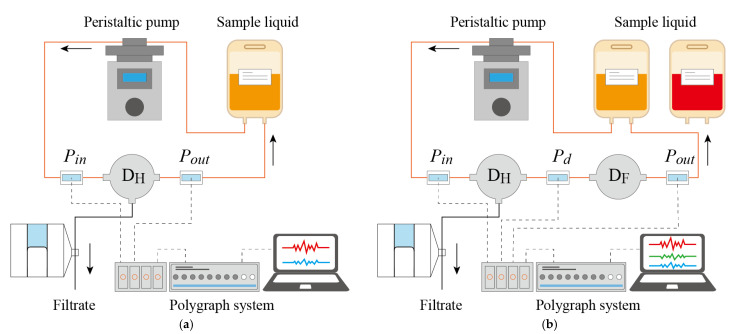
Perfusion system for in vitro experiments with *D_H_* alone (**a**) and with *D_H_* and *D_F_* (**b**). Pressures at the inlet and outlet of the device are measured with pressure transducers and a polygraph system. Pressure and flow rate are controlled with the peristaltic pump, while the tubes can be clamped or opened to ambient conditions.

Figure 5 shows the in vitro experimental setup: a perfusion circuit was designed to contain a peristaltic pump (Peri-Star Pro; World Precision Instruments, Sarasota, FL, USA), extension tubes (SF-ET2022L; Terumo Corporation, Tokyo, Japan), and hemofiltration devices *D_H_* and *D_F_*. Instead of blood, saline solution (Otsuka Pharmaceutical, Tokyo, Japan) with albumin (FUJIFILM Wako Pure Chemical Corporation) was used for samples. $P_{in}$ and $P_{out}$ were measured with pressure transducers (DX-100; Nihon Kohden Corporation, Tokyo, Japan) and a polygraph (RMT-1000MG; Nihon Koden Corporation, Tokyo, Japan) and controlled by the peristaltic pump. Filtrate was stored in a tube and the amount of filtrate was calculated from the movement of the front edge of the filtrate in the tube.

#### 2.4.3. Filtration Coefficient

We conducted in vitro experiments to obtain $L_{pf}$ and $L_{pbf}$ using the perfusion circuit. Saline solution with albumin was used as the sample liquid. The filtrate was stored inside a tube. The amounts of filtration and backfiltration were measured from the movement of the front edge of the filtrate. Oncotic pressure was deduced from Equation (3) and experimentally; first, the sample channel was clamped before storage. Since there was no flow in the channel, $P_{in}=P_{out}$. When no filtrate is obtained,$\;P_{c}=P_{in}=P_{out}$.

In obtaining $L_{pf}$, $P_{in}$ and $P_{out}$ were substituted into Equations (1) and (2). When $L_{pbf}$ was measured, backfiltration was intentionally induced by setting the saline solution channel open to ambient conditions after the filtrate channel was filled with filtrate. $TMP=-P_{c}$, then $L_{pbf}$ could be calculated from Equation (2). Using the obtained $L_{pf}$ and $L_{pbf}$, the amount of filtrate could be calculated from Equations (2)–(5) as a function of $C_{TP}$, $P_{in}$, and $P_{out}$. Albumin concentrations of 0, 1.8, 4.1, and 5.9 were tested.

#### 2.4.4. Effect of Fluid-Resistive Device *D_F_*

The number of saline solution channels in the hemofiltration device $N_{D_{H}\;}$ was set as 5, while that in the fluid-resistive device $N_{D_{F}}$ varied, as 1, 5, or 10. Pressure $P_{d}$, the pressure between *D_H_* and *D_F_*, and the pressure at the inlet of *D_H_* and at the outlet of *D_F_*, which can be approximated as $P_{a}$ and $P_{v}$, respectively, were measured when volumetric flow rates were 5, 10, and 15 mL/min. Theoretically, the ratio of the pressure drop, $\left(P_{d}-P_{v})/(P_{a}-P_{v}\right)$, can be expressed as:

(8) $$\frac{P_{d}-P_{v}}{P_{a}-P_{v}}=\frac{N_{D_{H}}}{N_{D_{H}}+N_{D_{F}}}$$

and 0.83, 0.50, and 0.33 for $N_{D_{F}}$ of 1, 5, and 10, respectively.

#### 2.4.5. Ex Vivo Experiments

We conducted ex vivo experiments using beagle dogs to confirm the effectiveness of the pressure control device and changes in filtrate volume over time. Blood pressure and oncotic pressure depend on the individual animal [21,22,23]. In the case of beagle dogs, blood pressure in the artery and vein are 50–90 mmHg and 5–10 mmHg, respectively. Oncotic pressure $P_{c}$ ranges from 10–30 mmHg. We used *D_H_* with 5 blood layers and *D_F_* with 5 layers, when the pressure between devices $P_{d}$ is considered to be larger than $P_{c}$.

Dogs were caged indoors, with room lights on from 08:00 to 20:00, the was temperature controlled to 22–26 °C, and relative humidity at 50–60%. The dogs were fed 250 g/day of dog chow (CD-5M; Clea Japan Inc., Tokyo, Japan). Water was provided ad libitum. Anesthesia was preceded by overnight food deprivation. Dogs received subcutaneous administration of a mixture of 0.05 mg/kg of atropine (Mitsubishi Tanabe Pharma Corporation, Osaka, Japan) and 0.5 mg/kg of droperidol (Droleptan Injection; Alfresa Pharma Corporation, Tokyo, Japan), followed by the intravenous administration of thiopental sodium (Ravonal; Mitsubishi Tanabe Pharma Corporation, Osaka, Japan) until effect. Anesthesia and analgesia were maintained via the inhalation of 2.0–3.5% sevoflurane (Mylan Seiyaku, Tokyo, Japan) with the aid of an artificial respirator and the intravenous infusion of remifentanil (0.01 mg/kg/h, Ultiva Intravenous; Janssen Pharma K.K., Tokyo, Japan), respectively. Subjects were placed on an operating table in lateral recumbency, and vital signs including non-invasive blood pressure were monitored with a monitoring system (BSM-3592; Nihon Kohden). All animal experiments were approved by the President and by the Institutional Animal Care and Use Committee (IACUC) of Tokyo Medical University and performed in accordance with institutional, science community, and national guidelines for animal experimentation.

**Figure 6:**
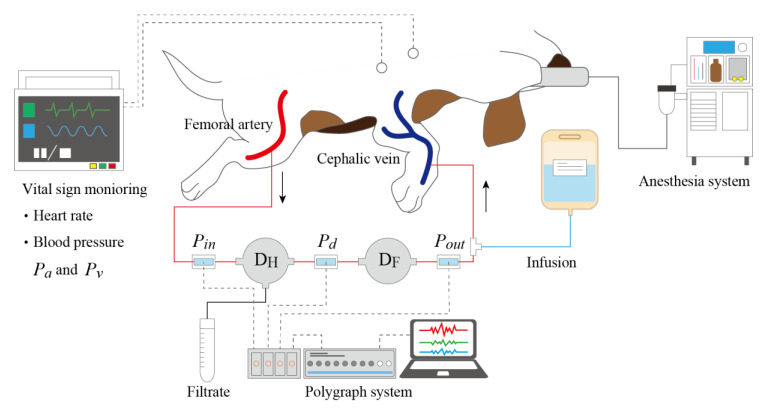
Setup for ex vivo experiments with beagle dogs. The inlet of the hemofiltration device was connected to the radial cutaneous vein of the foreleg and the outlet of the fluid-resistive device was connected to the femoral artery. Pressures at the inlets and outlets of devices were measured with transducers. Blood was collected to deduce the oncotic pressure after the experiments.

The processes for preparing *D_H_* and *D_F_* are detailed below. Saline was circulated using a peristaltic pump to remove air bubbles from the blood and filtrate layers. The devices were then placed in a container filled with saline to prevent drying and sterilized with gamma rays at 10 kGy [24]. Before starting the experiment, the hemodiafiltration device was connected to a pressure control device, a blood pressure monitor, and a 3.1 mm diameter extension tube, then a three-way stopcock (Terumo Corporation, Tokyo, Japan) was attached to both ends. The inside of the device was filled with a solution of 5000 units/5 mL of Na-heparin injection (Mochida Pharmaceutical Co. Ltd., Tokyo, Japan) mixed with saline in a ratio of 1:9. Beagle dogs were anesthetized by suction anesthesia, and the device was connected by puncturing the radial cutaneous vein of the foreleg and the femoral artery using an indwelling needle. The experimental setup is depicted in Figure 6. At the same time, 2 mL of blood was drawn from the arterial three-way stopcock using a syringe and heparin was administered from the venous three-way stopcock. The collected blood was transferred to a Venoject II vacuum blood collection tube (Terumo Corporation, Tokyo, Japan) for storage. After confirming that the device was filled with blood, filtrate collection and blood pressure monitoring were started. After completion of the experiment, 2 mL of blood was collected from the three-way stopcock on the arterial side using a syringe. The collected blood and filtrate were subsequently analyzed for albumin, total protein, urea nitrogen, and creatinine using a dry clinical chemistry analyzer (Spot-Chem EZ SP-4430; Arkray, Kyoto, Japan) and for electrolyte concentration using an electrode electrolyte analyzer (Spot-Chem EL SE-1520; Arkray, Kyoto, Japan). Total protein concentration was used to calculate the oncotic pressure.

## 3. Results

### 3.1. In Vitro Experiment

#### 3.1.1. Filtration Coefficient

**Figure 7:**
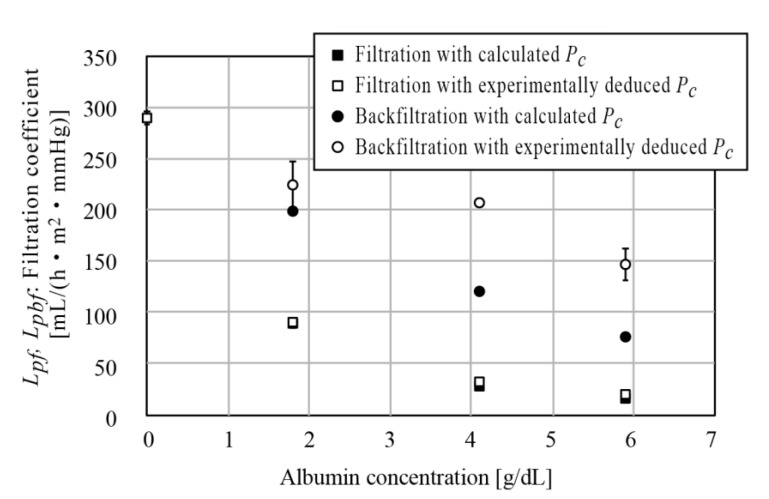
Filtration coefficients $L_{pf}$ and $L_{pbf}$ with respect to albumin concentration. The calculated $P_{c}$ and experimentally deduced $P_{c}$ had discrepancy, which led to the discrepancy particularly for $L_{pbf}$. Both coefficients decreased with albumin concentration due to fouling. $L_{pbf}$ was found to be larger than $L_{pf}$ and the discrepancy increased with increasing concentration.

The relationship between filtration coefficient and albumin concentration obtained is shown in Figure 7 (for date, see the supplemental material). The calculated $P_{c}$ and experimentally deduced $P_{c}$ showed discrepancies, which were found to particularly affect $L_{pbf}$. $L_{pbf}$ is larger than $L_{pf}$, which indicates that even small regions of negative *TMP* lead to reductions in filtrate. Both $L_{pf}$ and $L_{pbf}$ decreased with albumin concentration, which was considered to reflect fouling of albumin, as albumin molecules blocked some of the nanopores in the PES membrane. This was observed in our previous work [5,25]. Fouling only takes place on the saline channel side, since the albumin molecules are larger than the nanopores of the PES membrane and thus are not present in the filtrate. In the case of backfiltration, the filtrate returns from the filtrate channel to the saline channel, which can lead to alleviation of the fouling. This explains why $L_{pbf}$ was larger than $L_{pf}$ and the discrepancy increased with albumin concentration.

Since $L_{pbf}$ was several times larger than $L_{pf}$, when $P_{in}>P_{c}>P_{out}$, the resulting filtrate amount was small, as expected from Equation (4). The fluid-resistive device *D_F_* therefore plays a crucial role in increasing pressure at the outlet of the hemofiltration device *D_H_* to prevent backfiltration, even though the volumetric flow rate decreases given the blood pressure.

#### 3.1.2. Effect of Fluid-Resistive Device *D_F_*

**Figure 8:**
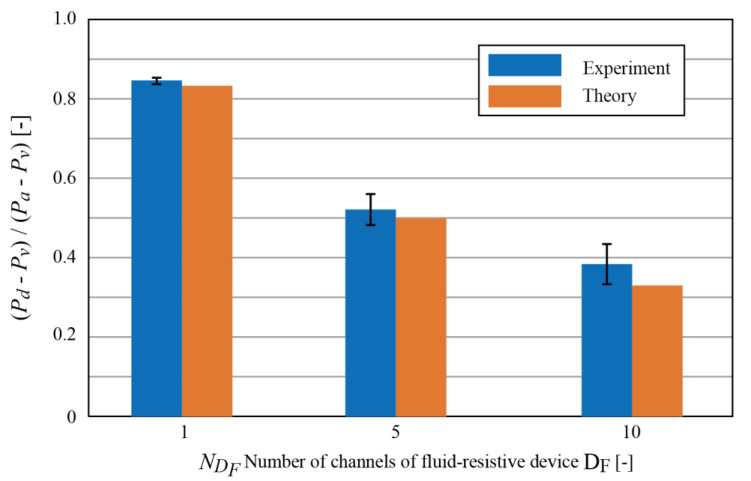
Ratio of the pressure drop in the hemofiltration device *D_H_* and the fluid-resistive device *D_F_* as a function of the number of the channels of *D_H_*. The theoretical value was deduced from Equation (8).

Figure 8 shows the ratio of pressure loss across the hemofiltration device *D_H_* to that across the fluid-resistive device *D_F_* (for date, see the supplemental material). The theoretical value was deduced from Equation (6). $N_{D_{H}}$ was 5 and $N_{D_{F}}$ varied between 1, 5, and 10. Volumetric flow rates of 5, 10, 15 mL/min were tested. Discrepancies between experimental and theoretical values were considered to originate from pressure losses at the inlets and outlets. Saline solution was introduced to the device with a tube, then divided into the channels. This causes a pressure loss and depends on the geometry and the number of channels.

The results proved the effectiveness of the fluid-resistive device. The fluid-resistive device needs to be designed such that the pressure inside the hemofiltration device is always larger than the oncotic pressure given the blood pressure and total protein concentration.

### 3.2. Ex Vivo Experiment

**Figure 9:**
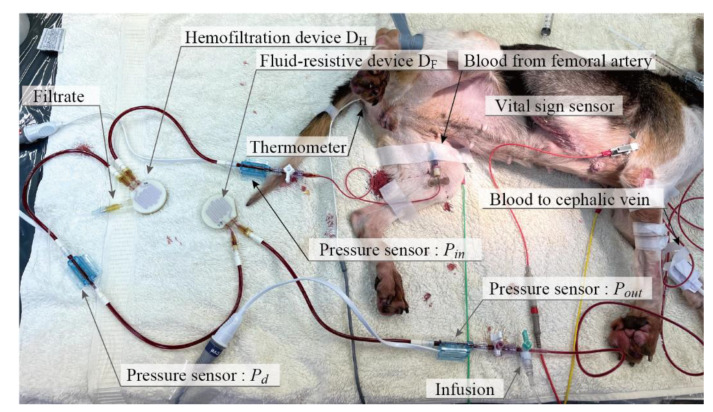
Photograph of an ex vivo experiment with a beagle dog. The animal is under anesthesia. The devices are connected to the femoral artery and cephalic vein. Filtrate is collected while pressures $P_{in}$, $P_{out}$, and $P_{d}$ are continuously measured. Vital signals including $P_{a}$ and $P_{v}$ are also recorded. Blood is collected to measure $C_{TP}$ and deduce $P_{c}$.

The photo of the experimental setup is shown in Figure 9 (for the schematic diagram, see Figure 6). We conducted three experiments for 90 min. We measured the amount of filtrate every 15 min. The detailed results are summarized in Table 1, Table 2 and Table 3.

As shown in Table 2, filtrate was continuously collected, and the amount decreased with time. $P_{in}$ was found to be lower than the mean arterial pressure by approximately 20 mmHg, due to pressure loss at the connection to the artery. $P_{out}$ was also larger than the venous pressure (typically 5–10 mmHg), originating from the pressure loss after the device. The pressure loss partially depends on the connection between the tubes and the blood vessels by puncturing. $P_{d}$ was successfully ramped up from $P_{out}$ by *D_F_*.

Table 3 shows the components of blood and filtrate. As designed, no albumin was found in the filtrate, while components of plasma were maintained in the filtrate. From the albumin concentration, oncotic pressure $P_{c}$ was 17.2, 13.9, and 16.1 mmHg for Subjects A, B, and C, respectively.

**Figure 10:**
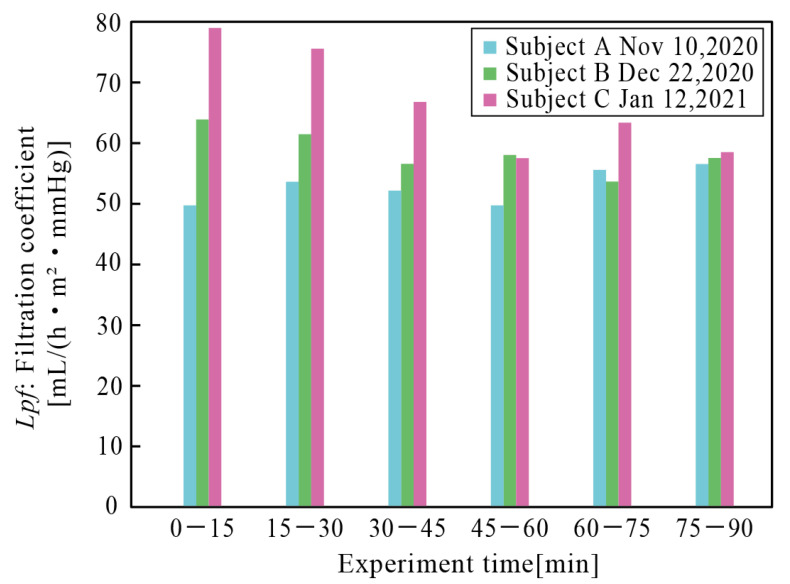
Filtration coefficients in the ex vivo experiments with respect to experiment time. Filtrate was collected every 15 min. No significant decreases or fluctuations were observed. Filtration coefficients are comparable to those from in vitro experiments with an albumin concentration of around 2 g/dL.

The filtration coefficient was deduced from the results over time, as shown in Figure 10. They ranged from 50 to 90, comparable with the results obtained in in vitro experiments (see Figure 7 when albumin concentration is around 2 g/dL). No severe effect of sterilization with gamma rays was found. A decrease in the filtration coefficient was observed for Subject C, which we attributed to fouling. For these three cases, no effect of blood coagulation was observed for 90 min. Longer experiments need to be conducted to further investigate the impacts of blood coagulation. However, since beagle dogs in ex vivo experiments needed to be anesthetized, the experiments were limited to several hours to prevent overdose. Since ex vivo experiments with awake animals are extremely challenging, in vivo experiments with implanted IHFDs are mandatory to study blood coagulation, and we will present these investigations in the near future.

## 4. Discussion

Backfiltration has been studied in hemodialysis applications, since backfiltration brings the waste material removed from the blood back to the blood and deteriorates the dialysis performance. In hemofiltration, the components of the filtrate are theoretically identical to those of blood plasma, but backfiltration decreases the amount of filtrate and, worse (as shown in Figure 7), the filtrate coefficient of backfiltration, $L_{pbf}$, was experimentally found to be several times larger than that of filtration, $L_{pf}$. Therefore, the region in which backfiltration takes place needs to be designed to be as small as possible.

The fluid-resistive device *D_F_* was designed to include only blood channels. The channel design was identical to that of the hemofiltration device *D_H_* for the ease of fabrication. By serially connecting *D_F_* after *D_H_*, the pressure drop across the two devices was divided as designed in both in vitro and ex vivo experiments. In the ex vivo experiments, pressure at the outlet of *D_F_* may be larger than the oncotic pressure $P_{c}$. If this is the case, even without *D_F_*, TMP inside *D_H_* is always positive and no backfiltration will take place. However, note that large pressure drops between $P_{a}$ and $P_{in}$ and between $P_{out}$ and $P_{v}$ were generated. This was caused by the extension tubes and indwelling needles that bridge the femoral artery and *D_H_* and the radial cutaneous vein of the forearm and *D_F_* [26]. Within vivo experiments, the IHFD will be placed in the vicinity of the artery and vein (in the case of humans, the internal iliac artery and external iliac vein will most likely be used [27,28]) and connected to vessels with minimum pressure loss. $P_{in}$ and $P_{out}$ will be close to $P_{a}$ and $P_{v}$, resulting in $P_{in}>P_{c}>P_{out}$. Therefore, the fluid-resistive device *D_F_* is necessary.

In the three experiments, for 90 min, no severe blood coagulation was observed. In the applications for IHFDs, the filtration capacity needs to be maintained over a period of years, so the long-term effects on blood coagulation need to be investigated. Since ex vivo experiments involve devices and tubes outside the animals, only animals under anesthesia can be tested and the period of experiments is limited to several hours due to the risk of overdose on anesthetic agents [29]. For in vivo experiments, device implantation in awake animals needs to be conducted to extend the experiment time.

## 5. Conclusions

In this paper, we described the design and testing of a hemofiltration device that works under blood pressure. Backfiltration caused by oncotic osmotic pressure was found to decrease the amount of filtrate and deteriorate the filtration capacity, which was even promoted by the larger filtration coefficient for backfiltration rather than that for positive filtration. The fluid-resistive device that is serially connected to the hemofiltration device was designed such that the output pressure of the hemofiltration device was larger than the oncotic pressure. Ex vivo experiments in beagle dogs for 90 min were successfully conducted. The findings from this study are now ready to be used to design devices for long-term in vivo experiments.

## Figures and Tables

**Table 1:** Basic information on the 3 beagle dogs (A, B, C).

		Subject A	Subject B	Subject C
Date of experiment		10 November 2020	22 December 2020	12 January 2021
Sex		Male	Female	Male
Age (years)		4.0	16.8	4.3
Body weight (kg)		13.2	5.6	11.2
Blood pressure (mmHg)	sBP	125	81	121
	dBP	51	57	55
	MAP	86	67	82

sBP: systolic blood pressure, dBP: diastolic blood pressure, MAP: mean arterial pressure.

**Table 2:** Pressure and amount of filtrate during the experiments.

**Subject A**				
**Time (min)**	**Filtrate (mL)**	$P_{in}$ **(mmHg)**	$P_{d}$ **(mmHg)**	$P_{out}$ **(mmHg)**
0–15	1.89	58.04	54.08	46.85
15–30	1.70	51.66	47.60	40.50
30–45	1.63	51.35	47.20	40.01
45–60	1.56	51.71	47.49	40.16
60–75	1.59	48.62	44.63	37.78
75–90	1.34	43.46	39.81	33.75
**Subject B**				
**Time (min)**	**Filtrate (mL)**	$P_{in}$ **(mmHg)**	$P_{d}$ **(mmHg)**	$P_{out}$ **(mmHg)**
0–15	1.53	36.38	33.33	25.35
15–30	1.41	35.32	32.49	24.47
30–45	1.39	36.93	34.00	25.62
45–60	1.43	36.92	33.94	25.66
60–75	1.35	37.37	34.39	26.04
75–90	1.44	37.20	34.21	26.11
**Subject C**				
**Time (min)**	**Filtrate (mL)**	$P_{in}$ **(mmHg)**	$P_{d}$ **(mmHg)**	$P_{out}$ **(mmHg)**
0–15	1.29	33.28	27.55	19.59
15–30	1.32	34.35	28.44	20.12
30–45	1.32	36.67	30.21	21.18
45–60	1.27	38.86	31.95	22.16
60–75	1.40	38.87	31.94	22.07
75–90	1.23	37.75	31.13	21.49

**Table 3:** Composition of blood and filtrate.

**Subject A**						
**Sample**	**Alb (g/dL)**	**BUN (mg/dL)**	**Cre (mg/dL)**	**Na (mmol/L)**	**K (mmol/L)**	**Cl (mmol/L)**
Blood (before experiment)	1.4	13	0.6	149	3.6	109
Blood (after experiment)	1.5	13	0.4	148	3.6	109
Filtrate (0–15)	- *	15	0.5	146	3.5	110
15–30	-	16	0.6	147	3.7	110
30–45	-	16	0.6	147	3.7	108
45–60	-	16	0.6	145	3.6	109
60–75	-	16	0.5	146	3.6	108
75–90	-	15	0.5	145	3.6	108
**Subject B**						
**Sample**	**Alb (g/dL)**	**BUN (mg/dL)**	**Cre (mg/dL)**	**Na (mmol/L)**	**K (mmol/L)**	**Cl (mmol/L)**
Blood (before experiment)	1.2	11	- **	133	3.5	112
Blood (after experiment)	-	13	-	138	3.4	112
Filtrate (0–15)	-	14	-	143	3.5	104
15–30	-	14	-	141	3.8	104
30–45	-	15	-	142	3.7	103
45–60	-	15	-	142	3.7	103
60–75	-	16	-	141	3.6	103
75–90	-	16	-	143	3.6	104
**Subject C**						
**Sample**	**Alb (g/dL)**	**BUN (mg/dL)**	**Cre (mg/dL)**	**Na (mmol/L)**	**K (mmol/L)**	**Cl (mmol/L)**
Blood (before experiment)	2.2	17	0.4	148	4.4	102
Blood (after experiment)	2.2	19	0.4	147	5.2	102
Filtrate (0–15)	-	13	-	144	3.9	106
15–30	-	24	0.5	146	3.9	106
30–45	-	23	0.5	144	4.2	107
45–60	-	25	0.5	145	4.4	107
60–75	-	24	0.5	146	4.5	106
75–90	-	25	0.5	144	4.6	103

Alb: albumin, BUN: blood urea nitrogen, Cre: creatine, Na: sodium, K: potassium, Cl: chloride. *, ** The detection limits of Alb and Cre are 1.0 g/dL and 0.2 mg/dL, respectively.

## Data Availability

Data are contained within the Supplementary Material. Supplementary material is available online at https://www.mdpi.com/article/10.3390/mi12080992/s1.

## Supplementary Materials

The following are available online at https://www.mdpi.com/article/10.3390/mi12080992/s1.
